# Monitoring activity of hip injury patients (MoHIP): a sub-study of the World Hip Trauma Evaluation observational cohort study

**DOI:** 10.1186/s40814-020-00612-2

**Published:** 2020-05-22

**Authors:** Laura C. Armitage, Yuan Chi, Mauro Santos, Beth K. Lawson, Carlos Areia, Carmelo Velardo, Peter J. Watkinson, Lionel Tarassenko, Matthew L. Costa, Andrew J. Farmer

**Affiliations:** 1 Nuffield Department of Primary Care Health Sciences, Radcliffe Primary Care Building, Radcliffe Observatory Quarter, University of Oxford, Woodstock Road, Oxford, OX2 6GG UK; 2 Institute of Biomedical Engineering, Department of Engineering Science, University of Oxford, Oxford, UK; 3 Nuffield Department of Clinical Neurosciences, University of Oxford, Oxford, UK; 4 Nuffield Department of Orthopaedics, Rheumatology and Musculoskeletal Sciences, University of Oxford, Oxford, UK

**Keywords:** Hip fracture, Rehabilitation, Activity, Monitoring

## Abstract

**Background:**

Hip fracture is common, affecting 20% of women and 10% of men during their lifetime. The trajectory of patients’ recovery as they transition from the acute hospital setting to their usual residence is poorly understood. Recently, the use of activity trackers to monitor physical activity during recovery has been investigated as a way to explore this trajectory.

**Methods:**

This prospective observational cohort study followed patients from hospital to home as they recovered from a hip fracture. Participants were recruited from a single centre and provided with a 3-axis logging accelerometer worn as a pendant, for 16 weeks from recruitment. Participants received monthly follow-up visits which included questions about wearing the monitor. Monthly activity monitor data were also downloaded. Participant activity was estimated from the monitor data using the calibrated “Euclidean Norm Minus One” (ENMO) metric. Polynomial mixed-effects modelling was used to evaluate the difference between the weekly activity trends of 2 groups of participants: those with and without independent mobility at 16 weeks (defined by whether aids or personal assistance were required to mobilise).

**Results:**

Twenty-nine participants from 125 eligible patients were recruited. Of these, 19 (66%) reported being aware of wearing the monitor at least some of the time. Fourteen (48%) participants withdrew before study completion. Data for thirteen (45%) participants were of sufficient quantity to be included in the activity modelling procedure. Of these, 8 reported independent mobility at 16 weeks post-surgery, and 5 did not. By week 7, the weekly predicted mean ENMO (${\overline{ENMO}}_W$) values were significantly different between the two participant groups, demonstrating feasibility of the model’s ability to predict which patients will report independent mobility at 16 weeks.

**Conclusions:**

This is the first study to our knowledge to investigate acceptability and feasibility of a pendant-worn activity monitor in this patient cohort. Acceptability of wearing the monitor and feasibility of recruitment and retention of participants were limited. Future research into the use of activity monitors in this population should use minimally intrusive devices which are acceptable to this population.

**Study registration:**

MoHIP is a sub-study of the World Hip Trauma Evaluation (WHiTE) Study (ISRCTN 63982700).

## Background

Hip fracture is a common injury, affecting 20% of women and 10% of men during their lifetime. In 2018, the James Lind Alliance identified the top two research priorities in fragility fracture of the lower limb as firstly understanding the best in-hospital and secondly best out-of-hospital physiotherapy regimes for recovery [1]. However, little is known about the trajectory of recovery in mobility as patients transition from hospital to home. More recently, the use of activity trackers to monitor motion and physical activity during recovery from hip fracture is being investigated as a way to explore this trajectory [2, 3].

In 2018, the UK National Hip Fracture Database Report revealed that just 38.4% patients receive follow-up of their mobility at 120 days post injury [4] and the UK ‘Hip Sprint’ audit revealed that only 20% of hospitals achieved continuity of rehabilitation as patients transitioned to home [5]. Most people who sustain a hip fracture will have completed their rehabilitation by 4 months [5]. Research investigating the recovery of mobility during hip fracture rehabilitation has traditionally relied upon patient self-report questionnaires, collected intermittently and at long intervals and as such are vulnerable to biases of self-report, recall and social desirability [6].

There is a need for more detailed and objective information concerning recovery of patient mobility following a hip fracture, particularly at the critical point of transition from hospital to home, where continuity of rehabilitative care is frequently not achieved. Small and lightweight activity monitors provide an opportunity to measure the recovery of mobility with greater objectivity and frequency in this population.

The overall aim of this study was to assess feasibility and acceptability of collecting individual-level activity data via a wearable activity monitor worn on a pendant, to better understand the recovery of mobility in patients during the first 4 months following hip fracture. Study objectives were as follows:

1. Investigate the acceptability of wearing the activity monitor for the duration of the 4-month follow-up period through participant self-report about wearing the monitor
2. Assess feasibility of using 3-axis logging accelerometers to collect activity data in this population by:
  - Assessment of participant recruitment
  - Assessment of participant retention rate
  - Investigating whether an algorithm can be developed and applied to analyse low-intensity raw activity data obtained from the monitors.
  - Investigating whether this can appropriately differentiate between those participants who report independent mobility versus requiring assistance with mobility at 16 weeks.

## Methods

### Study design

**Fig. 1:**
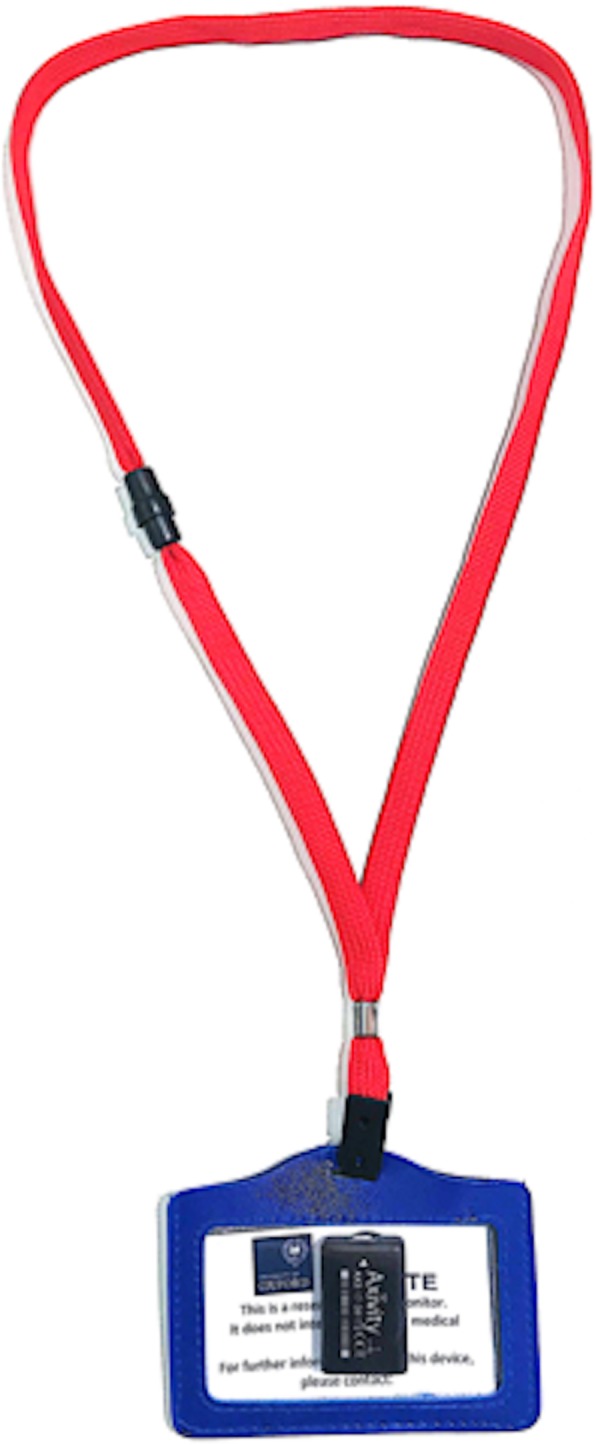
Axivity Ltd AX3 monitor fitted in a pendant

This prospective observational cohort study followed participants from hospital to home for 16 weeks as they recovered from a hip fracture injury. Participants were recruited from a single centre (John Radcliffe Hospital, Oxford, UK) from May 2018 to August 2019. At the point of recruitment, participants were provided with a 3-axis logging accelerometer, the AX-3 (Axivity Ltd, Newcastle upon Tyne, UK), worn within a pouch attached to a pendant as shown in Fig. 1. A pendant was chosen for two main reasons; firstly, with the monitor being capable of 3-axis data capture, we sought to investigate whether an algorithm could be developed to analyse low-intensity data with the monitor worn in the axial position, as previous studies have been limited to investigating this on an arm or leg [2, 3, 7]. Secondly, we aimed to investigate whether this would be an acceptable method of wear in an older population who are frequently familiar with personalised alarm systems [8] commonly worn as a pendant. Participants received a follow-up visit at their place of residence every 4 weeks for up to 16 weeks from recruitment. At each visit, a custom-made case report form was completed with the participant which included the following:

1. A 5-item mobility scale (Additional file 1)
2. A 3-item scale about awareness of wearing the activity monitor (Additional file 1)
3. A 5-item scale about how the participant felt about wearing the monitor (Additional file 1)

At all visits (except final), the participant was supplied with a replacement, fully-charged, monitor.

### Participants

#### Eligibility

Patients eligible for recruitment to MoHIP were those who met eligibility criteria for, and were recruited to, the World Hip Trauma Evaluation (WHiTE) cohort study [9]. WHiTE is an observational study in which patients receive usual medical and rehabilitative care and are also followed up to collect the UK Core Outcome Set for research into hip fracture [10]. Inclusion criteria were the same as for the WHiTE study (age ≥ 60 and receiving operative treatment for hip fracture) [9], and exclusion criteria specific to the MoHIP sub-study were as follows:

(i) Cognitive impairment
(ii) Discharged from hospital to a nursing home as permanent place of residence
(iii) Discharged to an area outside the hospital’s neighbouring counties of Berkshire, Buckinghamshire and Oxfordshire.

Patients with cognitive impairment were not eligible for inclusion owing to the potential safety implications that might arise from prolonged wear of a pendant system to house the activity monitor. Participants who were discharged to a temporary rehabilitation facility were eligible for inclusion and received the same research follow-up visits as those who were discharged to their own homes.

#### Consent

Eligible patients were approached at the first appropriate time during the post-operative period when they had regained capacity (typically the first day post-operatively). They were provided with study information including detailed information about the monitor, its capability, and how it would be worn. Participants were provided with the opportunity to ask questions and discuss the study with the researcher, their family, and carers for as long as required. All patients were required to give written consent to both the WHiTE study and MoHIP sub-study to participate.

### Data sources

Baseline data were collected for recruited participants including demographic data (age, sex), baseline comorbidities, pre-fracture mobility, type of fracture, type of anaesthesia, type of surgery and health status using the EuroQol 5-Dimension 5-Level measure (EQ-5D-5 L) [11].

#### Activity monitoring

Monitors were configured to record activity for 28 days at a sampling frequency of 25 Hz and range of ± 8 *g*. This configuration enabled the capture of the movements of interest in this study, most in the range up to 12 *Hz* [12]. The activity monitor was replaced at each 4-weekly follow-up visit by the primary care research nurse, and the monitor worn during the preceding month was returned to the study team for data download.

#### Outcome measures

The primary outcome measure was acceptability of using a CE-marked activity-monitoring device worn as a pendant, to assess participants’ physical activity during their recovery from a hip fracture. Acceptability was measured according to:

i. Participant wear time
ii. Percentage participants who reported feeling positive about wearing the monitor half or more of the time.

We assessed feasibility of using the activity monitor devices to estimate activity in this patient population through:

i. Participant recruitment rates
ii. Completion of study protocol

iii. Identifying patterns of activity extracted from monitor data, looking for correlations with standard core outcome measures and investigating whether the activity data can identify patients who are inactive. Participants were required to have activity data for ≥ 50% (≥ 8 weeks) of the study period to be included in this analysis.

A threshold of 80% is frequently considered an appropriate cut-off for assessing measures of acceptability and feasibility in pilot studies and was therefore adopted for this study [13]. For example, the wearing of the monitor could be considered acceptable if (i) ≥ 80% participants wore the monitor beyond 13 weeks of the 16-week study period (80% of the follow-up period) and (ii) ≥ 80% of participants reported feeling positive about wearing the monitor half or more of the time. Similarly, recruitment and retention of participants to this study could be considered feasible if (i) ≥ 80% eligible and approached participants were recruited and (ii) ≥ 80% of those recruited complete the study protocol.

#### Sampling and risk of bias

In order to limit sampling bias, all participants recruited to the WHiTE cohort study were screened for eligibility for MoHIP. Participants of all levels of baseline mobility and functional ability were screened for inclusion. Recruited participants were not provided with feedback regarding their activity levels, minimising risk of observation bias.

All participants recruited to the study received usual medical and rehabilitative care.

### Data management and statistical methods

This study was designed to investigate acceptability and feasibility of the use of a wearable activity monitor in an older and complex patient population. We have estimated that with a sample size of 29 participants, we would be able to detect a 20% withdrawal rate with a 95% confidence interval of ± 12%.

Study data were managed using REDCap electronic data capture tools hosted at the University of Oxford, providing a secure electronic data capture system [14, 15].

**Fig. 2:**
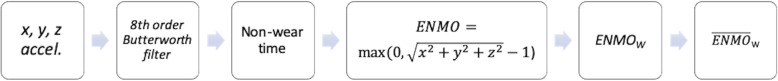
Block diagram of the pre-processing steps for each participant’s accelerometer data. ENMO is the calibrated “Euclidean Norm Minus One” metric. ENMO_W_ corresponds to each participant’s average EMNO per week. ${\overline{\mathrm{ENMO}}}_{\mathrm{W}}$ are predictions resulting from a mixed-effects model derived from the participants’ ENMO_W_ data

Figure 2 shows the block diagram of the accelerometer data pre-processing steps. Each participant’s data was first filtered using an eighth-order Butterworth bandpass filter, with cut-off frequencies of 0.2 *Hz* (highpass) and 12 *Hz* (lowpass) [7]. A non-wear time metric was then applied to remove periods in which the tracker was not being used. This metric was defined as all the non-overlapping 30-min windows in which the standard deviation (SD) was below 13 *mg*, in each of the three axes, or with an absolute value of less than 50 *mg* in at least 2 of the axes. Additionally, given that most participants did not wear the activity monitors during the night, only the accelerometer data recorded between 06:30 and 21:30 were considered.

The “Euclidean Norm Minus One” metric, $\mathrm{ENMO}=\max \left(0,\sqrt{x^2+{y}^2+{z}^2}-1\right)$, was used as a proxy for the patient activity [7, 16–18], in which *x*, *y*, and *z* represent each of the axes of the accelerometer sensor; the standard gravity created at the Earth’s surface (1 *g*) is subtracted; and negative ENMO values are set to zero. The latter may be a result from calibration errors, noise or negligible body movement [16]. The ENMO (computed at the sensor’s 25 Hz sampling rate) was averaged per minute, and then per week (from 1 to 16 post-operative weeks), for each participant. Each participant’s average ENMO per week is denoted as ENMO_W_ thereafter.

#### Mixed-effects model

A polynomial mixed-effects model was used to analyse the association between the participants’ ENMO_W_ (dependent variable) and the number of post-operative recovery weeks and the mobility outcome, i.e. those with and without independent mobility (defined by whether aids or personal assistance were required to mobilise at 16 weeks). The latter was modelled as a fixed-effect (binary variable denoted “mobility” coded 1 for participants reporting independent mobility and 0 otherwise). Random-effects were considered at the participant level for the number of post-operative weeks variable (coded as “week”). A normal prior was considered for both the random-effects terms and for the model error term, and the model fit achieved by maximum likelihood estimation. The model polynomial order was varied from 1 to 5, and the cubic model was found to have both the lowest Akaike and Bayesian Information Criteria. The latter is named “baseline model” (${\overline{\mathrm{ENMO}}}_W$) thereafter, with the formula (in Wilkinson notation):

$$ {\overline{\mathrm{ENMO}}}_W=1+\mathrm{mobility}+\mathrm{wee}\mathrm{k}+\mathrm{wee}{\mathrm{k}}^2+\mathrm{wee}{\mathrm{k}}^3+\left(\mathrm{week}+\mathrm{wee}{\mathrm{k}}^2+\mathrm{wee}{\mathrm{k}}^3|\ \mathrm{patient}\right). $$

#### Statistical analysis

The Likelihood Ratio Test (LRT) was used to assess if the baseline model with an additional clinically relevant parameter (added as an interaction or an additional fixed-effects term) achieved a significantly better model fit. We identified three additional parameters for investigation. Firstly, receipt of physiotherapy in the first month post hospital discharge (coded as 1 for or out-of-hospital physiotherapy and 0 otherwise) which was selected due to the nationally low reported rates of continuity of physiotherapy upon discharge [5] and previously reported negative association of lack of post discharge physiotherapy on functional recovery [19]. Secondly we investigated the effect of self-report of anxiety or depressive symptoms at baseline assessment on model fit as this has previously been shown to be negatively associated with regain of physical independence following hip fracture [20]. Finally, we investigated the effect of baseline self-report of pain and discomfort during the hospital stay on the model as this has been previously shown to be negatively associated with both ability to mobilise in the early post-operative period [21] and functional regain of outdoor mobility post hospital discharge [22].

## Results

### Participants

#### Enrolment

**Fig. 3:**
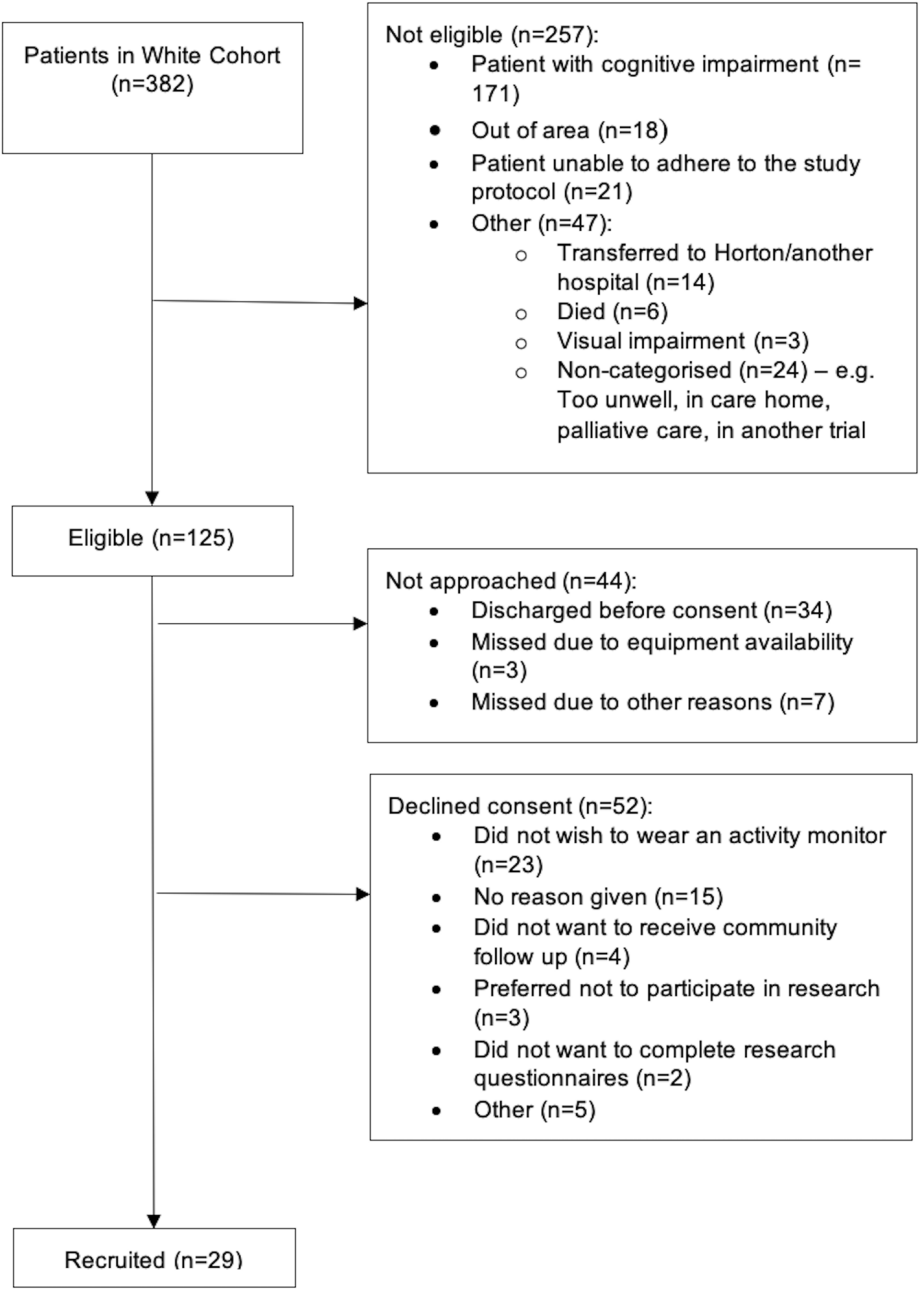
Participant screening and enrolment flow diagram

Between May 2018 and August 2019, 125 of 382 participants recruited to the WHiTE Cohort Study were eligible for recruitment to MoHIP. The most common reason for exclusion was cognitive impairment (*n* = 171, 44.7%). Of the eligible 125 participants, 81 (65%) were approached and 29 (36%) of these were recruited to MoHIP, meaning that the study did not meet the recruitment feasibility threshold of enrolling ≥ 80% of those eligible and approached. The flow diagram of screening and enrolment is shown in Fig. 3. Participant characteristics at baseline of the whole cohort and the thirteen participants included in the activity data modelling are presented in Table 1.

**Table 1:** Participant characteristics

			Recruited Cohort (*n* = 29 unless otherwise stated)	*n* = 13 (activity monitor group)
Age, median [IQR]			76 [71, 82]	77 [71, 84]
Women, *n* (%)			18 (64.3)	12 (92.3)
Pre-admission residence, n (%)	Own home		28 (100)	13 (100)
	Nursing home		0 (0)	0 (0)
Pre-fracture mobility	Freely mobile without aids		22 (78.6)	9 (69.2)
	Mobile outdoors with one aid		4 (14.3)	3 (23.1)
	Mobile outdoors with 2 aids or a frame		1 (3.6)	0 (0)
	Some indoor mobility, only mobilise outdoors with help		1 (3.6)	1 (7.7)
Type of fracture, *n* (%)	Intracapsular		19 (67.9)	9 (69.27)
	Extracapsular		8 (28.6)	4 (30.7)
	Sub-trochanteric		1 (3.6)	0 (0)
Type of surgery, n (%)	Arthroplasty—total hip replacement		10 (35.7)	5 (38.4)
	Hemiarthroplasty		6 (21.4)	2 (15.4)
	Fixation	Sliding hip screw	6 (21.4)	2 (15.4)
		Cannulated screw	2 (7.1)	2 (15.4)
		IM nail (long)	4 (14.3)	2 (15.4)
Type of anaesthesia, *n* (%)	General		20 (71.4)	11 (84.6)
	Intra-operative sedation		9 (32.1)	2 (15.4)
	Intra-operative nerve block		11 (39.3)	7 (53.8)
	Spinal		10 (35.7)	3 (23.1)
Medical history, *n* (%)	Myocardial infarction		2 (7.1)	2 (15.4)
	Heart failure		1 (3.6)	1 (7.7)
	Stroke/TIA		2 (7.1)	2 (15.4)
	Diabetes		0 (0)	0 (0)
	Renal dysfunction (moderate to severe)		0 (0)	0 (0)
	Respiratory disease		5 (18.5)	3 (23.1)
	Rheumatoid arthritis or connective tissue disease		3 (10.7)	2 (15.4)
	Malignancy		10 (35.7)	4 (30.8)
ASA Score, *n* (%)	I		3 (10.7)	1 (7.7)
	II		12 (42.9)	5 (38.5)
	III		6 (21.4)	2 (15.4)
	IV		5 (17.9)	3 (23.1)
	V		0 (0)	0 (0)
	Unknown		2 (3.7)	2 (15.4)
Other clinical factors	Receipt community physiotherapy in 1st month		8 (28.6)*	3 (23.1)
	Pain/discomfort (baseline)*	None	13 (48.1)	6 (46.2)
		Slight	6 (22.2)	4 (30.8)
		Moderate	8 (29.6)	3 (23.1)
		Severe	0 (0)	0 (0)
		Extreme	0 (0)	0 (0)
	Anxiety/depression (baseline)*	None	19 (70.4)	11 (84.6)
		Slight	6 (22.2)	2 (15.4)
		Moderate	1 (3.7)	0 (0)
		Severe	0 (0)	0 (0)
		Extreme	1 (3.7)	0 (0)

*ASA Score* American Society of Anesthesiologists physical classification system score [23]

*Data available for 28 participants

### Feasibility and acceptability of using the pendant-worn activity monitor for data collection

#### Participant retention and experience of wearing the monitor

Of the 29 recruited participants, 15 (52%) wore the activity monitor for 13 weeks or more, but two of these participants had data missing for ≥ 50% (8 weeks) of the study period and were therefore ineligible for inclusion in the activity data modelling procedure. The study therefore did not achieve the feasibility thresholds of ≥ 80% participants wearing the monitor for ≥ 80% of the follow-up period. Of the 29 participants, 19 (68%) reported being aware of the monitor at least some of the time. Fourteen (48%) participants withdrew before study completion. Reasons for withdrawal broadly related to two themes: firstly, participants reported the pendant system “got in the way” or was too visible; secondly, some participants reported that ongoing study participation involving daily monitor use and monthly visits was too high a demand in the context of an already high medical burden.

Of the 15 participants who completed the study protocol, 2 (13%) reported being unaware of the monitor during periods of wear, and 9 reported being aware of the monitor at least some of the time; 8 of these participants reported a negative awareness such as the monitor getting in the way, being too visible or irritating them. However, 7 (50%) of the 14 participants who completed the study protocol reported feeling positive or very positive about wearing the monitor at half or more of their follow-up visits, and 3 participants reported feeling positive or very positive about wearing the monitor throughout the study period.

Overall, participant wear of the Axivity monitor in the pendant system as defined for this study did not meet pre-defined thresholds for feasibility and acceptability.

#### Activity data analysis

The participants started wearing monitors after a median of 2 (IQR 1.8, 5) days post-surgery and were discharged from the hospital after a median of 6 (IQR 4, 10.5) days post-surgery. Only four participants had complete 16-week ENMO data, whilst 2 participants had half or more data missing and were excluded from the mixed-effects modelling. Thirteen patients (median age 77.4 [IQR 70.5, 83.7] years, 92.3% female) were included in the modelling procedure; 9 reported independent mobility at 16 weeks post-surgery, and the remaining 4 reported reliance on mobility aids or personal assistance. Demogpaphics for all participants and those who were included in the modelling analysis are presented in Table 1.

**Fig. 4:**
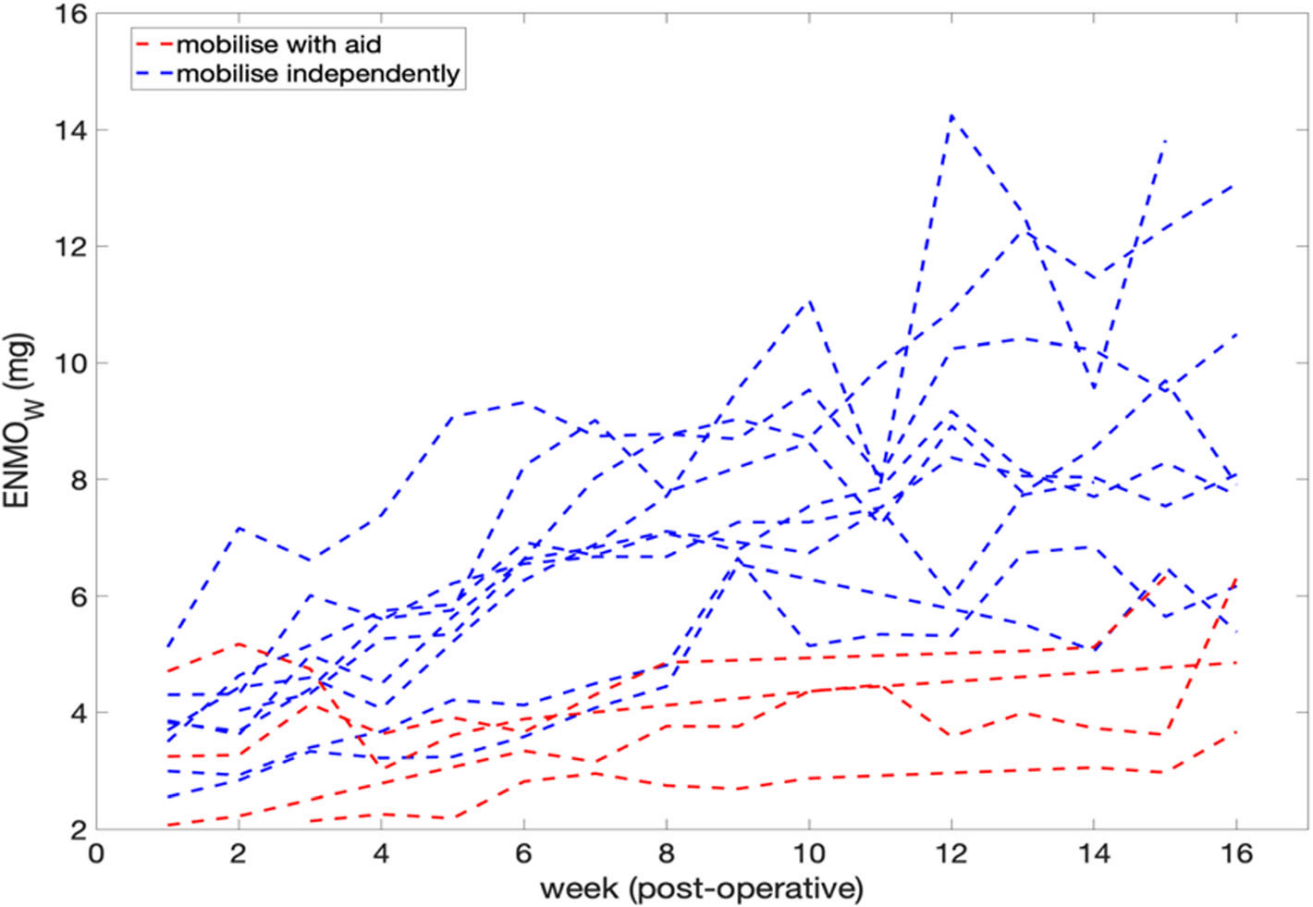
Average ENMO per post-surgery week for each patient (ENMO_W_). Blue and red represent the participants groups with and without independent mobility at 16 weeks, respectively

**Fig. 5:**
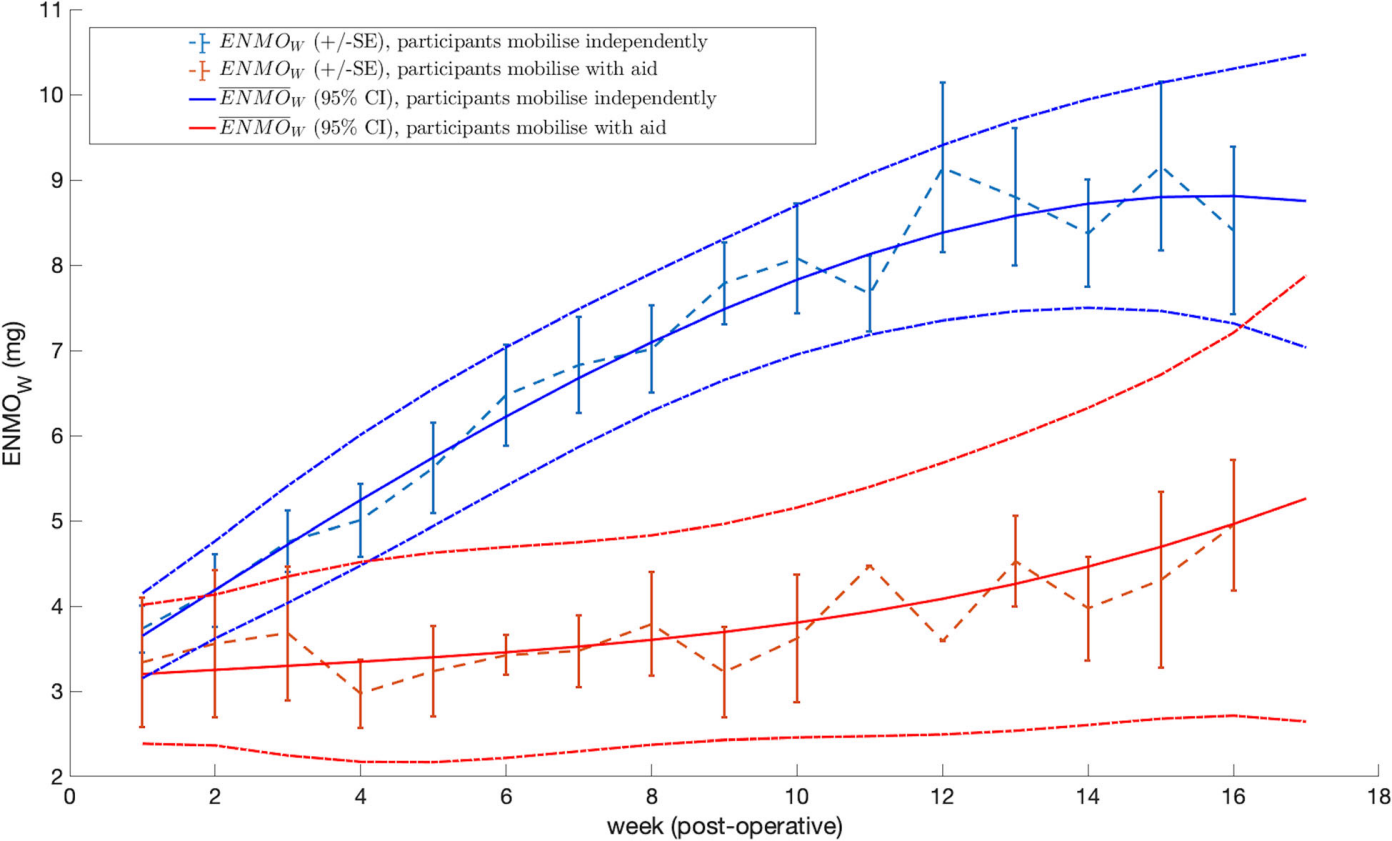
Linear fixed effects model: polynomial fit (*n* = 3) from 6.30 am to 9.30 pm. The average ENMO_W_ ± SE per week are shown for each mobility outcome group in dashed lines. The ${\overline{\mathrm{ENMO}}}_{\mathrm{W}}$(and 95% CI) predictions of the mixed-effects model are also shown for each group in solid lines. Groups with and without independent mobility at 16 weeks are coloured blue and red, respectively

Each participant’s ENMO_W_ recovery trend is shown in Fig. 4 (in blue and red). It can be observed that very little overlap exists between the groups from week 4, with those who self-reported mobilising independently at 16 weeks showing a much higher ENMO_W_ from week 4 onwards (a participant reaching a maximum ENMO_W_ of 13 *g* versus 6 *g* in each group). The average ENMO_W_ ± SE is presented in Fig. 5 in dashed lines, for each mobility outcome group. A significant increase in the average ENMO_W_, from 3.7 *g* (3.5, 4.0 SE) to 8 *g* (7.4, 9.4 SE), can be seen for the independently mobile group, versus an average ENMO_W_ increase from 3.3 *g* (2.6, 4.1 SE) to 5.0 *g* (4.2, 5.7 SE, significantly lower), for the aid-dependent mobility group, from 1 to 16 post-operative weeks.

#### Mixed-effects model

Figure 5 shows the ${\overline{ENMO}}_W$ (and 95% Confidence Interval, CI) in solid lines, predicted from the baseline model, for each mobility outcome group. When considering data grouped at the participant level, the ${\overline{ENMO}}_W$ is significantly different between the two groups (i.e. the 95% CI do not overlap) from week 5. The overlap at week 16 is caused by the lower amount of accelerometer data available in the latter weeks. This model indicates that accelerometer data can inform clinical staff about which patients are experiencing a faster or slower recovery in their mobility.

Finally, using the LRT statistic, it was verified that none of the other clinical factors (receipt of physiotherapy in the community post hospital discharge, pain/discomfort and anxiety/depression evaluated at the baseline questionnaire) provided a better model fit when added to the baseline model.

## Discussion

### Key results

#### Feasibility

We recruited 29 participants to this study, which assessed feasibility of using a pendant-worn activity monitor to better understand the recovery of mobility in patients during the first 4 months of recovery post-hip fracture injury. We observed low consent rates and a high withdrawal rate. However, this study is novel in its collection of meaningful activity data through an axial-worn activity monitor in this patient cohort, with previous studies being limited to monitors worn on the wrist or thigh [2, 3, 7]. By week 5, the predicted ${\overline{\mathrm{ENMO}}}_W$ values were significantly different between the two mobility outcome groups (independently mobile versus not) meaning the model was able to inform the mobility outcome at 16 weeks. This identifies a potential opportunity for increased rehabilitative input for those demonstrating less activity at weeks 4–5 of recovery, with the aim of improved mobility outcomes at 16 weeks.

Whilst we established feasibility of the monitor’s ability to capture meaningful activity data when worn in this way, feasibility of using such a monitor for future research or clinical practice is limited by the limited feasibility of recruitment and retention of participants, as well as acceptability of the monitor observed in this study. The high withdrawal rate (48%), coupled with some reports of a negative awareness of the monitor from those who completed the study protocol, demonstrates that the method of wearing the monitor employed in this study is not optimally suited to this demographic of the population. This adds an important contribution to the currently limited literature regarding optimal sensor placement and method of wear [24].

### Limitations

This observational study excluded the large proportion of the hip fracture population who have concomitant cognitive impairment. The decision to exclude this group of patients was made owing to the potential safety implications that might arise from prolonged wear of a pendant system to house the activity monitor. The exclusion of this patient group limits the generalisability of our findings. However, whilst it is known that dementia is known to be a risk factor for poorer outcomes after hip fracture [25], there is some evidence to suggest that cognitive impairment does not have a significant impact on functional regain if rehabilitation is received [26, 27]. It should be noted that the median age of recruited participants (76, IQR 71, 82) was also markedly lower than the median age of the European hip fracture population (82, IQR 75, 87) [28].

Further limitations include the number of participants that we were able to recruit (29 participants) and the number of those with sufficient accelerometer data available to generate an activity trend model (13 participants). Participant self-report of mobility was used to define which participants were independently mobile and which were reliant on aids or assistance. Self-report is vulnerable to bias, and the study may have been improved by the inclusion of a mobility assessment, performed by a healthcare professional. However, an observational study of over 88,000 non-institutionalised older adults observed a concordance rate of 80% between self-report and professional assessment of mobility [29]. Results of statistical tests evaluating the significance of the addition of clinical factors to the baseline model should be interpreted cautiously, as the ENMO_W_ dataset may be too small to derive conclusions from the LRT. The availability of data may have been further impaired by the design of the monitor.

In summary, although we were able to demonstrate a significant difference in the average activity between the group that gained full mobility at 16 weeks and the group that did not, this model is not generalisable to the full cohort of patients undergoing hip-fracture surgery, and could potentially be an overfitting to these data. Future work should evaluate its predictive performance on a held-out dataset.

## Conclusions

This is the first study to our knowledge to investigate acceptability and feasibility of a pendant-worn activity monitor in this patient cohort. We have demonstrated that the ENMO metric may serve as a proxy for the level of physical activity of hip fracture patients during their post-operative recovery period. However, feasibility of recruitment, retention of participants and acceptability of the method of wearing the monitor in this study were notably limited. We have therefore not been able to demonstrate that this specific approach to monitoring activity of hip fracture patients would be feasible for larger-scale research studies. Further research into the use of activity monitors in this patient population should use minimally intrusive devices which are acceptable and safe for a more inclusive population, such as those with cognitive impairment.

## Supplementary information

**Additional file 1.** Appendices.

## Data Availability

The datasets generated and analysed during the current study are not publicly available as further analyses and modelling of the data are currently being developed for publication, but datasets are available from the corresponding author on reasonable request.
