# Supplementary material for: Monitoring activity of hip injury patients (MoHIP): a sub-study of the World Hip Trauma Evaluation observational cohort study
Source: Pilot Feasibility Stud. 2020 May 22;6:70. doi: 10.1186/s40814-020-00612-2 (PMC7243330; doi:10.1186/s40814-020-00612-2)
Supplement: Supplementary file 1 — Additional file 1. Appendices. [file 40814_2020_612_MOESM1_ESM.docx]

**APPENDICES**

**Monitoring activity of Hip Injury Patients (MoHIP): An sub-study of the World Hip Trauma Evaluation Observational Cohort Study**

Laura C Armitage, Yuan Chi, Mauro Santos, Beth K Lawson, Carlos Areia, Carmelo Velardo, Peter J Watkinson, Lionel Tarassenko, Matthew L Costa, Andrew J Farmer.

**Appendix 1**

**
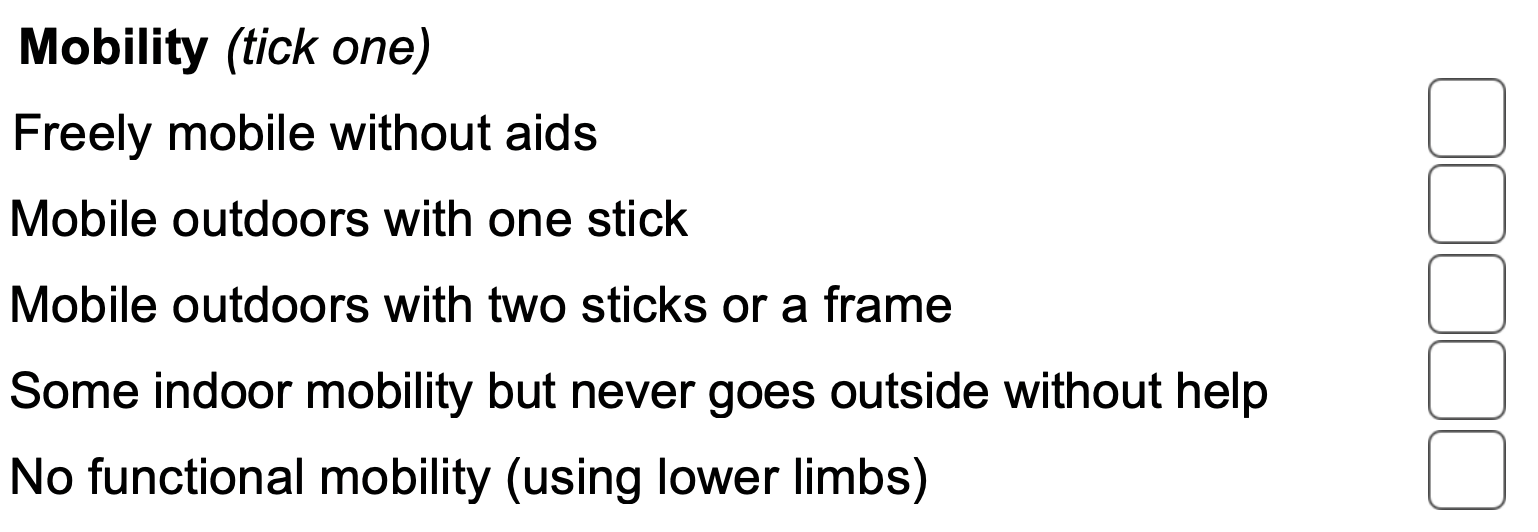
**

**Appendix 2**

**
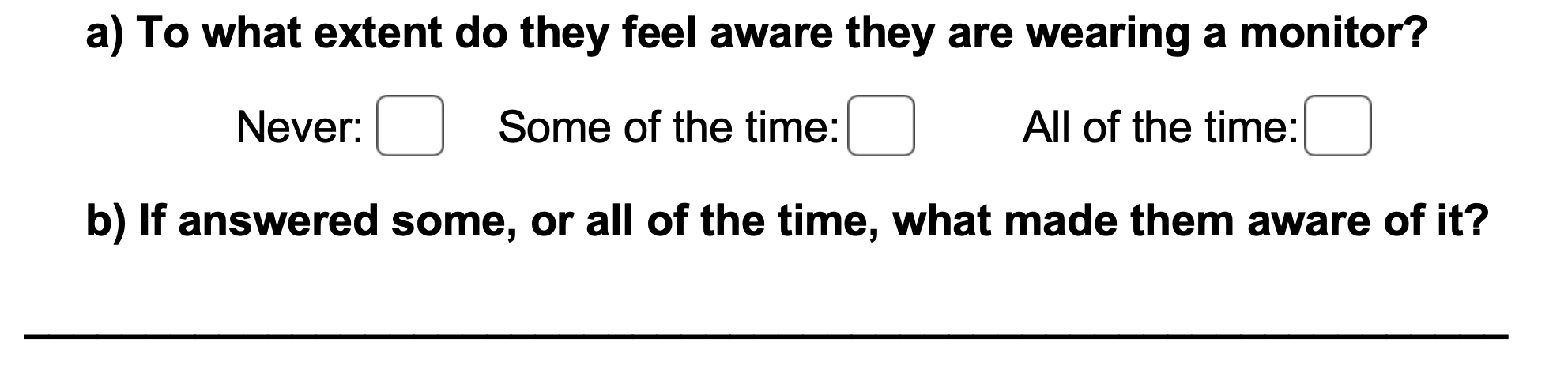
**

**Appendix 3**

**
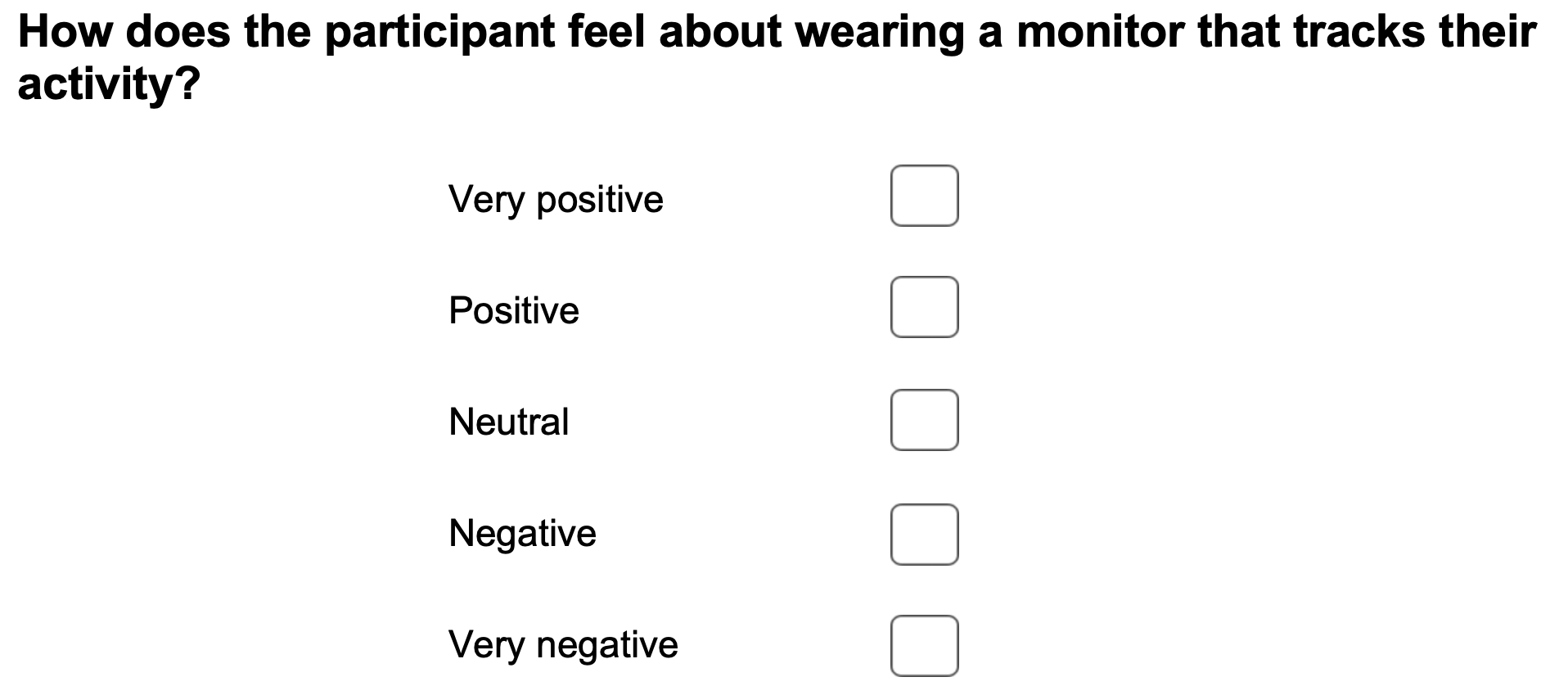
**
